# Supplementary material for: A simulation study of the use of temporal occupancy for identifying core and transient species
Source: PLoS One. 2020 Oct 23;15(10):e0241198. doi: 10.1371/journal.pone.0241198 (PMC7584212; doi:10.1371/journal.pone.0241198)
Supplement: S3 Fig — Percent of biologically core (A) species that were incorrectly inferred to be transient and biologically transient (D) species that were incorrectly inferred to be core for each combination of detection probability and landscape similarity at a broader dispersal kernel (99% of movements result in dispersal distances ≤ 8 grid cells). The x-axis is the average species detection probability for the simulation run, while the y-axis is the proportion of a 7 x 7 landscape surrounding the focal pixel that is of the same habitat type. Line graphs (B, E) show the percent of incorrect classifications of core species (B) or transient species (E) for each detection probability at low (0.3, solid line) or high (0.8, dashed line) landscape similarity. Line graphs (C, F) show the percent of incorrect classifications of core species (C) or transient species (F) with increasing landscape similarity at low (0.1, solid line) or high (0.9, dashed line) detection probability. (DOCX) [file pone.0241198.s003.docx]

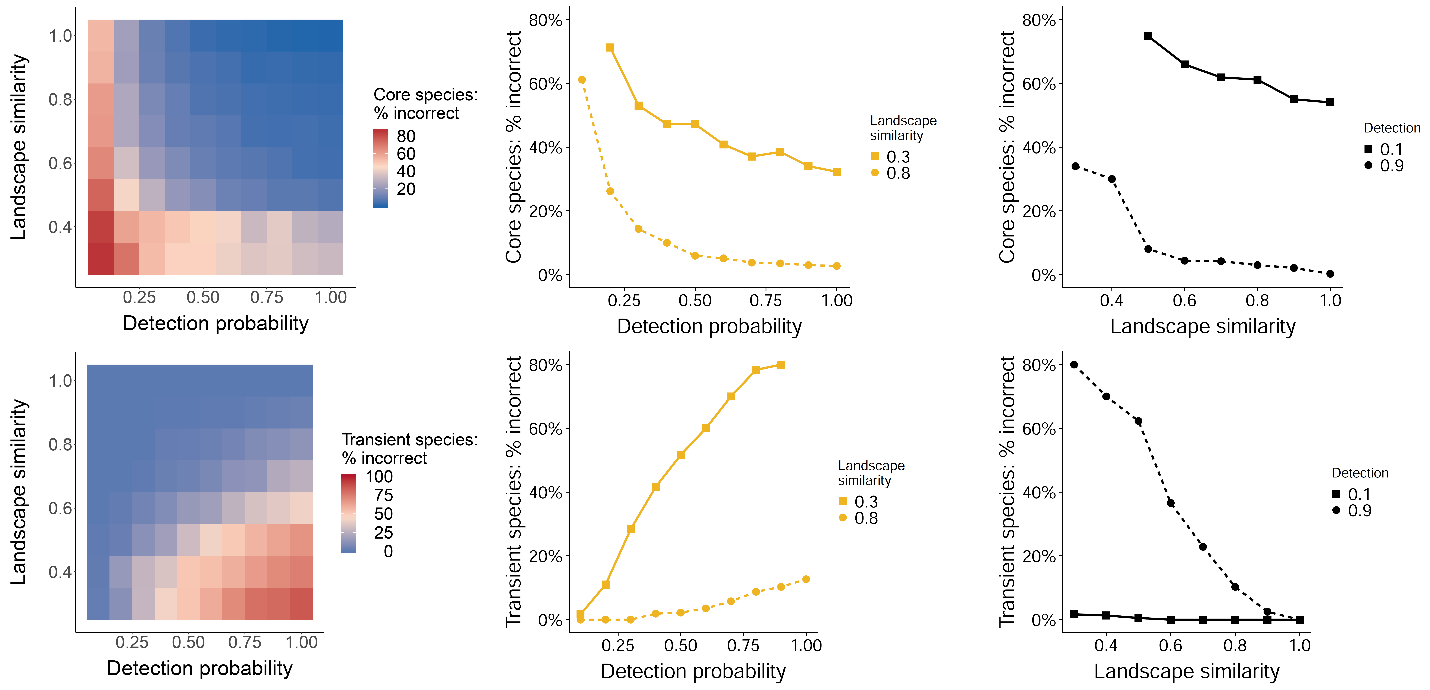


**S3 Fig.** Percent of biologically core (A) species that were incorrectly inferred to be transient and biologically transient (D) species that were incorrectly inferred to be core for each combination of detection probability and landscape similarity at a broader dispersal kernel (99% of movements result in dispersal distances ≤ 8 grid cells). The x-axis is the average species detection probability for the simulation run, while the y-axis is the proportion of a 7 x 7 landscape surrounding the focal pixel that is of the same habitat type. Line graphs (B, E) show the percent of incorrect classifications of core species (B) or transient species (E) for each detection probability at low (0.3, solid line) or high (0.8, dashed line) landscape similarity. Line graphs (C, F) show the percent of incorrect classifications of core species (C) or transient species (F) with increasing landscape similarity at low (0.1, solid line) or high (0.9, dashed line) detection probability.
