# Supplementary material for: A simulation study of the use of temporal occupancy for identifying core and transient species
Source: PLoS One. 2020 Oct 23;15(10):e0241198. doi: 10.1371/journal.pone.0241198 (PMC7584212; doi:10.1371/journal.pone.0241198)
Supplement: S4 Fig — (DOCX) [file pone.0241198.s004.docx]

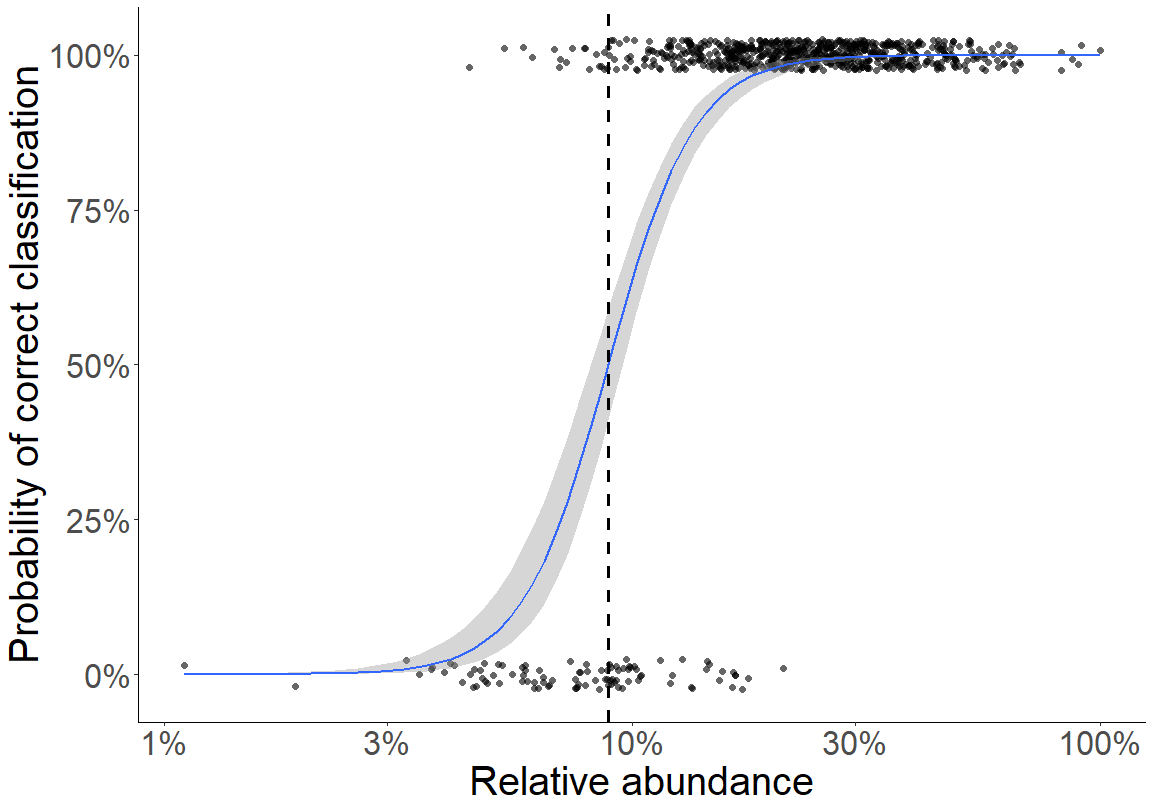


**S4 Fig.** Correct or incorrect classification of biologically core species based on temporal occupancy as a function of the log of landscape wide abundance (relative to the abundance of the most abundant species) at a broader dispersal kernel (99% of movements result in dispersal distances ≤ 8 grid cells).
