# Supplementary material for: A simulation study of the use of temporal occupancy for identifying core and transient species
Source: PLoS One. 2020 Oct 23;15(10):e0241198. doi: 10.1371/journal.pone.0241198 (PMC7584212; doi:10.1371/journal.pone.0241198)
Supplement: S7 Fig — Mean number of biologically core (A) and biologically transient (D) species observed for each combination of detection probability and landscape similarity at a broader dispersal kernel (99% of movements result in dispersal distances ≤ 8 grid cells). Line graphs (B, E) show the mean count of core species (B) or transient species (E) for each detection probability at low (0.3, solid line) or high (0.8, dashed line) landscape similarity. Line graphs (C, F) show the mean count of core species (C) or transient species (F) with increasing landscape similarity at low (0.1, solid line) or high (0.9, dashed line) detection probability. (DOCX) [file pone.0241198.s007.docx]

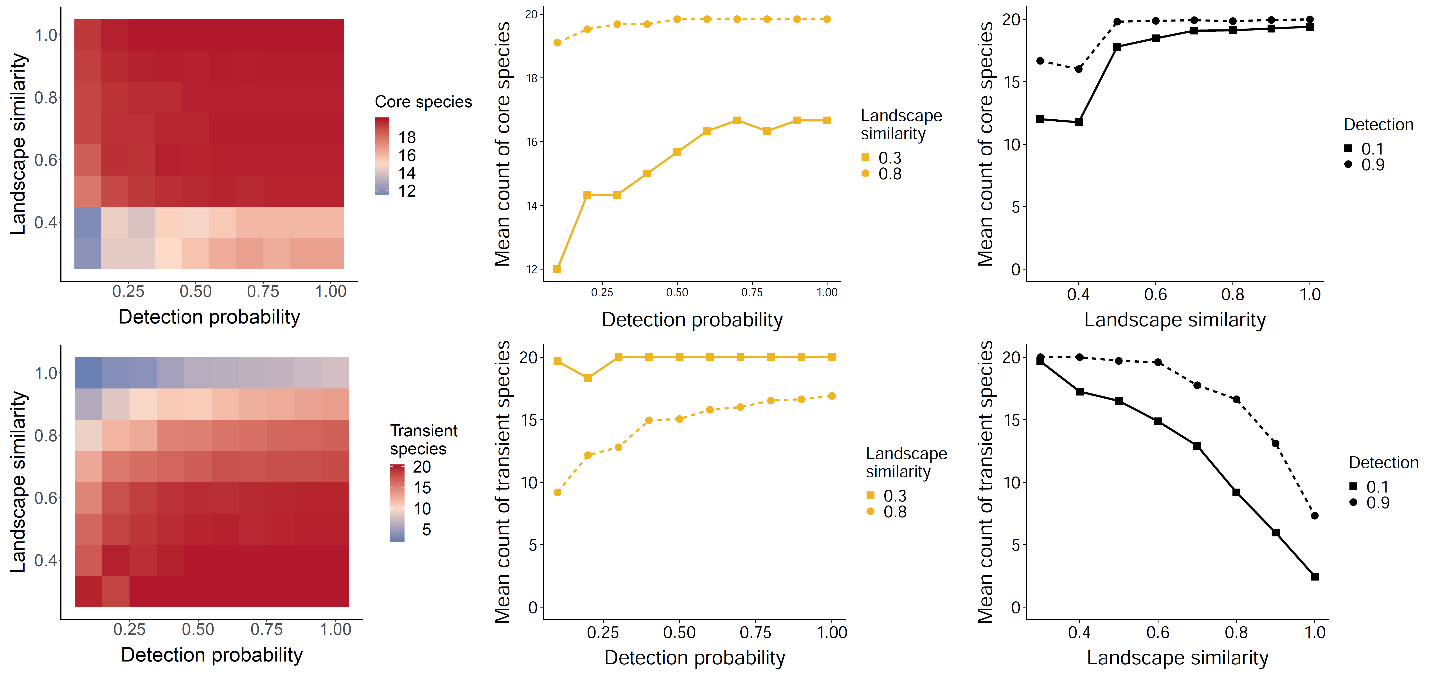


**S7 Fig.** Mean number of biologically core (A) and biologically transient (D) species observed for each combination of detection probability and landscape similarity at a broader dispersal kernel (99% of movements result in dispersal distances ≤ 8 grid cells). Line graphs (B, E) show the mean count of core species (B) or transient species (E) for each detection probability at low (0.3, solid line) or high (0.8, dashed line) landscape similarity. Line graphs (C, F) show the mean count of core species (C) or transient species (F) with increasing landscape similarity at low (0.1, solid line) or high (0.9, dashed line) detection probability.
