# Supplementary material for: A simulation study of the use of temporal occupancy for identifying core and transient species
Source: PLoS One. 2020 Oct 23;15(10):e0241198. doi: 10.1371/journal.pone.0241198 (PMC7584212; doi:10.1371/journal.pone.0241198)
Supplement: S1 Table — (DOCX) [file pone.0241198.s008.docx]

**S1 Table.** Parameter estimates from linear models of the number of core and transient species as a function of detection and landscape similarity for all dispersal kernels.

|  | Estimate | Standard Error | t-value | p-value |
| --- | --- | --- | --- | --- |
| ***dispersal kernel 4*** |  |  |  |  |
| core intercept | 10.54 | 0.17 | 61.39 | <2e-16 |
| core detection | 1.72 | 0.15 | 11.54 | <2e-16 |
| core landscape similarity | 8.02 | 0.19 | 41.76 | <2e-16 |
| transient intercept | 27.81 | 0.28 | 97.69 | <2e-16 |
| transient detection | 5.29 | 0.25 | 21.04 | <2e-16 |
| transient landscape similarity | -27.14 | 0.34 | -80.50 | <2e-16 |
|  |  |  |  |  |
| ***dispersal kernel 2*** |  |  |  |  |
| core intercept | 10.54 | 0.17 | 61.39 | <2e-16 |
| core detection | 1.72 | 0.15 | 11.54 | <2e-16 |
| core landscape similarity | 8.02 | 0.19 | 41.76 | <2e-16 |
| transient intercept | 23.32 | 0.34 | 69.05 | <2e-16 |
| transient detection | 4.08 | 0.32 | 12.84 | <2e-16 |
| transient landscape similarity | -24.63 | 0.42 | -58.16 | <2e-16 |
|  |  |  |  |  |
| ***dispersal kernel 8*** |  |  |  |  |
| core intercept | 16.62 | 0.19 | 88.64 | <2e-16 |
| core detection | 0.77 | 0.15 | 5.26 | <2e-16 |
| core landscape similarity | 3.20 | 0.21 | 15.40 | <2e-16 |
| transient intercept | 28.59 | 0.53 | 53.99 | <2e-16 |
| transient detection | 5.26 | 0.43 | 12.20 | <2e-16 |
| transient landscape similarity | -25.10 | 0.60 | -41.67 | <2e-16 |
