# Supplementary material for: A simulation study of the use of temporal occupancy for identifying core and transient species
Source: PLoS One. 2020 Oct 23;15(10):e0241198. doi: 10.1371/journal.pone.0241198 (PMC7584212; doi:10.1371/journal.pone.0241198)
Supplement: S2 Table — (DOCX) [file pone.0241198.s009.docx]

**S2 Table.** Parameter estimates from linear models of the percent incorrect core and transient species as a function of detection and landscape similarity for all dispersal kernels.

|  | Estimate | Standard Error | t-value | p-value |
| --- | --- | --- | --- | --- |
| ***dispersal kernel 4 (primary analyses)*** |  |  |  |  |
| core intercept | 0.54 | 0.01 | 46.87 | <2e-16 |
| core detection | -0.36 | 0.01 | -36.05 | <2e-16 |
| core landscape similarity | -0.18 | 0.01 | -13.69 | <2e-16 |
| transient intercept | 0.20 | 0.01 | 15.02 | <2e-16 |
| transient detection | 0.22 | 0.01 | 17.01 | <2e-16 |
| transient landscape similarity | -0.35 | 0.02 | -20.51 | <2e-16 |
|  |  |  |  |  |
| ***dispersal kernel 2*** |  |  |  |  |
| core intercept | 0.54 | 0.01 | 46.87 | <2e-16 |
| core detection | -0.36 | 0.01 | -36.05 | <2e-16 |
| core landscape similarity | -0.18 | 0.01 | -13.69 | <2e-16 |
| transient intercept | 0.20 | 0.01 | 15.02 | <2e-16 |
| transient detection | 0.22 | 0.01 | 17.01 | <2e-16 |
| transient landscape similarity | -0.35 | 0.02 | -20.51 | <2e-16 |
|  |  |  |  |  |
| ***dispersal kernel 8*** |  |  |  |  |
| core intercept | 0.67 | 0.02 | 40.26 | <2e-16 |
| core detection | -0.46 | 0.01 | -35.39 | <2e-16 |
| core landscape similarity | -0.34 | 0.02 | -18.57 | <2e-16 |
| transient intercept | 0.44 | 0.01 | 32.91 | <2e-16 |
| transient detection | 0.22 | 0.01 | 20.10 | <2e-16 |
| transient landscape similarity | -0.62 | 0.02 | -40.76 | <2e-16 |
